# Macrophage cells secrete factors including LRP1 that orchestrate the rejuvenation of bone repair in mice

**DOI:** 10.1038/s41467-018-07666-0

**Published:** 2018-12-05

**Authors:** Linda Vi, Gurpreet S. Baht, Erik J. Soderblom, Heather Whetstone, Qingxia Wei, Bridgette Furman, Vijitha Puviindran, Puviindran Nadesan, Matthew Foster, Raymond Poon, James P. White, Yasuhito Yahara, Adeline Ng, Tomasa Barrientos, Marc Grynpas, M. Arthur Mosely, Benjamin A. Alman

**Affiliations:** 1 Department of Orthopaedic Surgery, Duke University, Durham, 27710 USA; 2 Hospital for Sick Children, University of Toronto, Toronto, M5G1X8 Canada; 3 Duke Molecular Physiology Institute, Duke University, Durham, 27701 USA; 4 Proteomics and Metabolomics Shared Resource, Duke University, Durham, 27701 USA; 5 Mount Sinai Hospital, University of Toronto, Toronto, M5G1X5 Canada; 6 Regeneration Next, Duke University, Durham, 27710 USA; 7 Division of Physical Medicine and Rehabilitation, University of Toronto, M5G 2C4, USA

## Abstract

The pace of repair declines with age and, while exposure to a young circulation can rejuvenate fracture repair, the cell types and factors responsible for rejuvenation are unknown. Here we report that young macrophage cells produce factors that promote osteoblast differentiation of old bone marrow stromal cells. Heterochronic parabiosis exploiting young mice in which macrophages can be depleted and fractionated bone marrow transplantation experiments show that young macrophages rejuvenate fracture repair, and old macrophage cells slow healing in young mice. Proteomic analysis of the secretomes identify differential proteins secreted between old and young macrophages, such as low-density lipoprotein receptor-related protein 1 (Lrp1). Lrp1 is produced by young cells, and depleting *Lrp1* abrogates the ability to rejuvenate fracture repair, while treating old mice with recombinant Lrp1 improves fracture healing. Macrophages and proteins they secrete orchestrate the fracture repair process, and young cells produce proteins that rejuvenate fracture repair in mice.

## Abstract (web summary)

The rate of repair declines with age; however, exposure to young circulations can rejuvenate fracture repair, but how this is accomplished is unknown. Here, the authors identify proteins, including low-density lipoprotein receptor-related protein 1 (Lrp1), as being secreted from young macrophages and rejuvenating fracture repair in mice.

## Introduction

Tissue repair and regenerative capacity declines with age. The pace of fracture repair slows after skeletal maturity, with 3-month-old-juvenile mice (equivalent of an young adult) healing almost twice as fast as 20-month-old mice (equivalent to a 70 years old)^1^. Four weeks following fracture, a time in which callus from fractures in young animals contain a high proportion of bone, there will still be substantial proportions of undifferentiated mesenchymal or fibrous tissue in older mice. Many factors are proposed to slow the pace of fracture repair in older animals, including intrinsic changes in mesenchymal cells and hormonal changes with aging^2–4^. A smaller proportion of undifferentiated mesenchymal cells differentiate to osteoblasts in older animals, and this block to differentiation is one factor responsible for the delay in fracture healing in aging^5–8^. Parabiosis and bone marrow transplantation studies show that young hematopoietic cells can rejuvenate of the pace of fracture repair in old mice. Furthermore, conditioned media experiments show that secreted factors can increase the proportion of cells differentiating to osteoblasts in older animals^9^. Whereas this data are consistent with the notion that a secreted factor produced by hematopoietic cells can rejuvenate the pace of fracture repair, neither the hematopoietic cell type, nor the factors are known.

One cell type that might be responsible for the rejuvenation effect of young hematopoietic cells is a cell of the monocyte/macrophage lineage. After tissue injury, macrophages are recruited to areas of trauma, where they undergo phenotypic and functional changes coordinating tissue repair^10^. During fracture healing macrophages are found at the fracture site, and when depleted, fractures will not heal effectively^11,12^. Macrophage populations and phenotype can change with aging^13,14^.

Here we investigate the possibility that monocyte/macrophage lineage cells rejuvenate fracture repair. We show that young macrophage cells produce factors that promote osteoblast differentiation in bone marrow stromal cells. Heterochronic parabiosis and bone marrow transplantation studies show that young macrophages rejuvenate fracture repair, and old macrophage cells slow healing in young mice. Comparison of secretomes between old and young cells identifies differential secreted proteins, one of which is low-density lipoprotein receptor-related protein 1 (Lrp1). Depleting *Lrp1* abrogates the ability to rejuvenate fracture repair, whereas treating old mice with recombinant Lrp1 improves fracture healing.

## Results

### Young macrophages promote osteoblastic differentiation

**Fig. 1:**
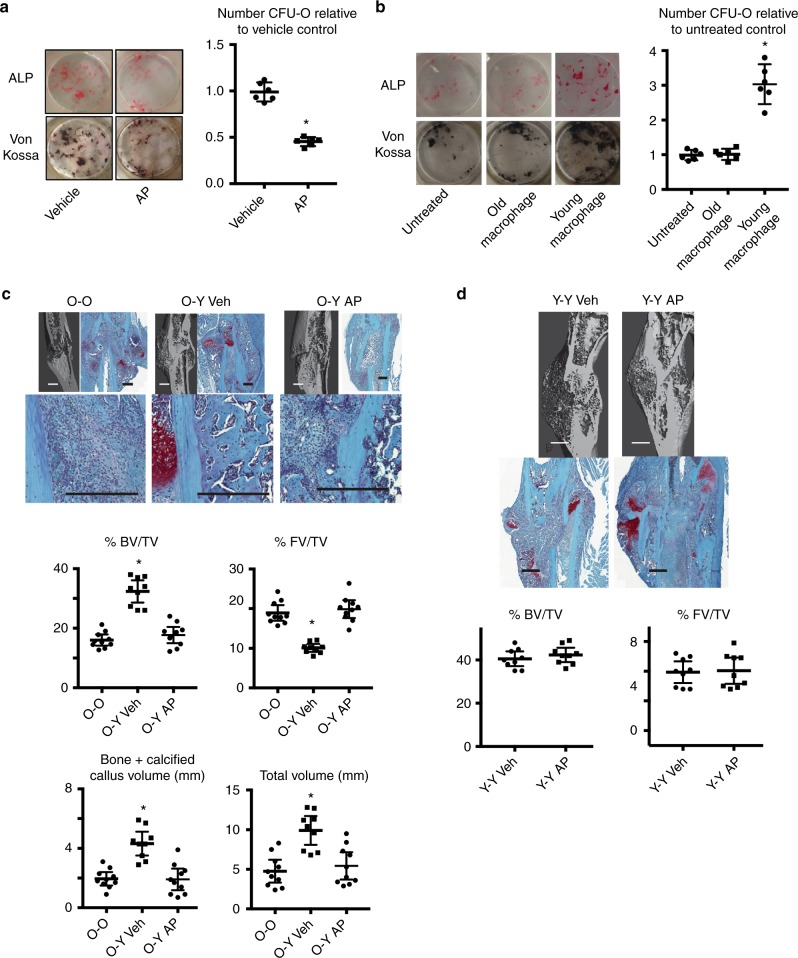
Young macrophage cells rejuvenate osteogenic differentiation and fracture repair. **a** Numbers of CFU-O (Von Kossa and Alk Phosphate (ALP) staining shown) that form when treated with conditioned media from the young bone marrow cell cultures from MaFIA mice when macrophages are depleted by AP20187 treatment (labeled AP) or when treated with a vehicle (Veh). Representative cell culture plates (left) and each data point is shown as well as means and 95% confidence intervals for data for each experimental group (right). An asterisk over a data point shows a significance *p* < 0.05 compared to controls with control culture defined as “1,” *n* = 6, two-tailed *t*-test. **b** Conditioned media from isolated macrophages from bone marrow from young, but not old mice increases CFU-O numbers that form from bone marrow cells obtained from old mice. Representative cell culture plates (left) and each data point as well as means and 95% confidence intervals for each experimental group with control culture normalized to “1” (right) are shown, *n* = 6, ANOVA. **c** Representative radiographic and Safranin O stained histologic images from the tibia of an old mouse 14 days after the bone was fractured in heterochronic parabiotic pairs in which the young parabiot was a MaFIA mouse (O-Y), or in isochronic parabiotic pairs in which both are old mice (O-O, *n* = 10). The MaFIA mouse pairs were treated with AP20187 (labeled AP, *n* = 10) to deplete macrophages from the young parbiot or vehicle (Veh, *n* = 9). Graphs show bone volume/total volume (BV/TV) in %; total fibrous tissue/total volume (FV/TV) in %; bone plus calcified callus volume (mm^3^), total callous volume (mm^3^), or relative density. Each data point is shown as well as means and 95% confidence intervals. **d** Similar data from young isochronic parabiotic pairs in which one mouse was a MaFIA mouse, showing that treatment with AP20187 did not alter normal tibia fracture healing, *n* = 9 in both groups. An asterisk over a data point indicates a significance *p* < 0.05 compared to controls, comparison using ANOVA (**c**) and two-tailed *t*-test (**d**)

Previous studies found that conditioned media from bone marrow cell populations that adhere to plastic, but not the non-adherent cell population from young animals increased the CFU-O capacity of older animals^9^. One cell type that is in the adherent cell population is the macrophage. Macrophage Fas-induced apoptosis transgenic mice (MaFIA) can be used to deplete macrophage cells. This mouse expresses a mutant human FK506 binding protein 1A, 12 kDa (FKBP12) driven by the mouse *Csf1r*, (colony stimulating factor 1 receptor) promoter. Expression is limited to macrophages, and when the mice are treated with the dimerizing reagent, AP20187, this induces apoptosis in macrophages^15^. Five days of treatment effectively depletes 90% of macrophage cells. Macrophage cells from these mice also express GFP prior to their depletion. We performed conditioned media experiment using bone marrow cells from young (4 months old) MaFIA mouse to rejuvenate osteogenic differentiation in bone marrow cells from old (20 months old) mice. When the cell cultures were treated with AP20187, to deplete the macrophage population, the rejuvenation effect on osteoblastic differentiation was lost (Fig. 1a). We then isolated macrophages from bone marrow from young or old mice, and found that conditioned media from young, but not old macrophage cells, would increase the capacity of old bone marrow cells to form CFU-O (Fig. 1b).

We next undertook parabiosis experiments in which we deleted macrophage cells from young (4 months old) mice in the parabiont pair and examined healing in the old (20 months old) mouse. Young or old MaFIA mice were anastomosed to old mice. After 3 weeks, a time in mice effectively share blood supply, half of the paired mice were treated with AP20187 to deplete macrophage lineage cells. The treatment was continued for the duration of the study. A week following this, one tibia from the old mouse was fractured as previously reported^16^. Two weeks later (the maximum duration our animal care committee would allow us to observe these animals) the mice were killed, and fractures analyzed for healing using micro-CT analysis and histomorphometry. We verified sharing of the blood supply and effective depletion of 90% of macrophage cells in the various animals at the time of killing using flow analysis (Supplementary Fig. 1). In these pairs, the ablation of the macrophages from the young parabiont abrogated the rescue effect of the youthful circulation on fracture healing in the old mice (Fig. 1c). In contrast, the ablation of macrophages from one mouse in isochronic pairs had a negligible effect on fracture healing (Fig. 1d). Osteoclasts have an effect on the ultimate state of a fracture. Previous investigation of fracture healing in MaFIA mice shows a mild effect of ablation of *Csf1r* expressing cells on osteoclast number and function^11^, a stark contrast to data from targeted disruption of the *Csf1r* gene, which results in osteopetrosis. Because one parabiont has not had ablation of macrophages, the effect of treatment with AP20187 should be further blunted. In support of this, the number of osteoclasts was not found to differ between the animals in the experimental groups (Supplementary Fig. 2).

### Young F4/80-positive cells promote healing in old mice

**Fig. 2:**
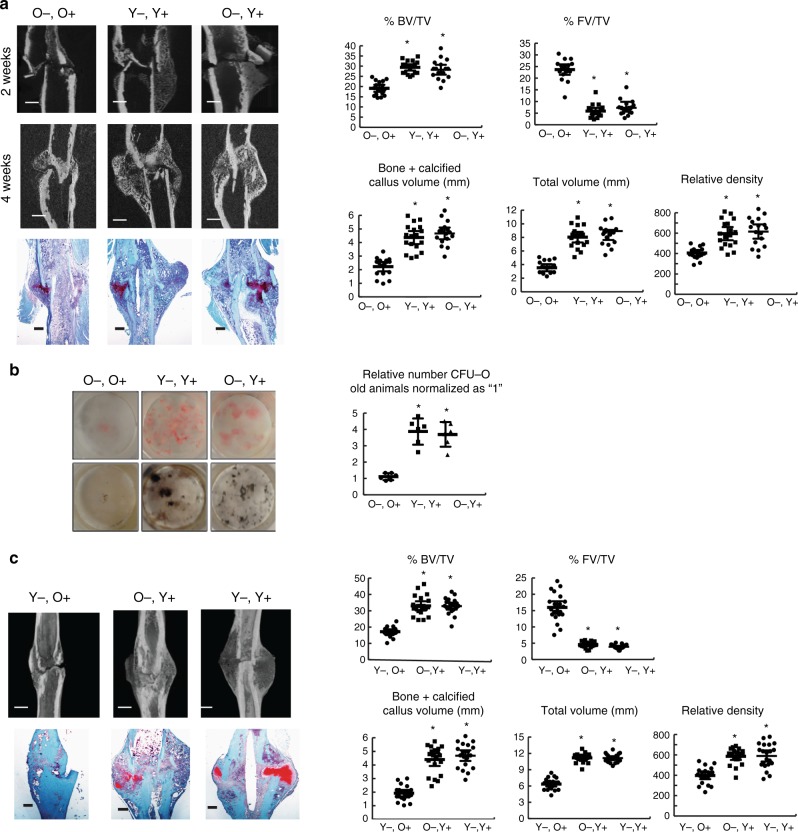
F4/80-positive cells from young animals enhance fracture repair and osteogenic differentiation. **a** Young (Y) and old (O) bone marrow cells were sorted into F4/80 high (+) and low (−) populations using flow cytometry and re-introduced into irradiated recipient old mice. Representative micro-CT radiographic images after a tibia fracture and Safranin O stained histologic images from the tibia of an old mouse 4 weeks after the bone was fractured in mice with transplanted with combinations of F4/80 high or low fractions from bone marrow from old or young donor mice. Graphs show bone volume/total volume (BV/TV) in %; total fibrous tissue/total volume (FV/TV) in %; bone plus calcified callus volume (mm^3^) or total callous volume (mm^3^), or relative density from tibias 4 weeks after fracture. Each data point is shown, along with means and 95% confidence intervals for each experimental group. *n* = 8 males, 9 females O−,O+, *n* = 9 males, 9 females for Y−,Y+, *n* = 9 males, 8 females for O−,Y+. **b** CFU-O from mice in A, with numbers that form from bone marrow cells from old mice. Representative cell culture plates (Von Kossa and Alk Phosphate staining shown) and each data point as well as means and 95% confidence intervals for each group with the mean from control culture defined as “1” (right). **c** Young (Y) and old (O) bone marrow cells sorted into F4/80 high (+) and low (−) populations using flow cytometry were re-introduced into irradiated recipient young mice (*n* = 6 in each group). Representative radiographic images 2 and 4 weeks after the fracture, and histologic images from the tibia of old mice 4 weeks after the bone was fractured transplanted with various combinations of F4/80 high or low bone marrow fractions. Graphs show each data point for bone volume/total volume (BV/TV) in %; total fibrous tissue/total volume (FV/TV) in %; bone plus calcified callus volume (mm^3^), total callous volume (mm^3^), or relative density. Data are shown as means and 95% confidence intervals. *n* = 11 males, 10 females for Y−;O+, *n* = 10 males, 10 females for O−:Y+, *n* = 10 males, 11 females for Y−;Y+. An asterisk over a data point shows a significance *p* < 0.05 compared to controls, comparison using ANOVA

To determine whether young macrophage/monocyte cell populations alone can improve fracture repair in older mice, we undertook bone marrow transplantation studies using fractionated donor cells. Macrophage populations from such transplants can survive for several months in the host animal^17^. Young and old bone marrow cells were sorted into F4/80 high (F4/80+) and low (F4/80−) populations using flow cytometry and re-introduced into irradiated recipient mice. We found equal numbers of transplanted macrophage cells at the fracture sites of these mice in all of the various combinations (Supplementary Fig. 3), showing effective engraftment and that the number of macrophage cells did not vary with the age of the donor or the age of the recipient. By sorting F4/80 positive and negative cells from the recipient mice that were transplanted with GFP expressing macrophage cells, we could identify if any recipient macrophage cells remained in the animals. We did not identify cells that did not express GFP, confirming that the cells in the mice were from the donor animals. Transplantation of old F4/80− with young F4/80+ cells improved fracture healing in these older animals relative to when old mice were transplanted with old F4/80− with old F4/80+ cells. Old animals that received young F4/80+cells displayed increased fracture callus calcification relative to animals that received old F4/80+ cells at 2 and 4 weeks following a fracture (Fig. 2a and supplementary data Figs. S4A and B). Cultures of bone marrow cells from animals engrafted with young F4/80+ cells showed increased osteoblast differentiation and matrix mineralization (Fig. 2b). There were equal numbers of F4/80-positive cells in each experimental group (Supplementary Fig. 4C). Furthermore, when old bone marrow cells were transplanted into young mice, there was a delay in fracture healing, recapitulating the effects of aging on fracture healing. Yet, when these young animals were engrafted with old F4/80− and young F4/80+ cells, they showed similar repair to animals engrafted with young F4/80− and young F4/80+ cells (Fig. 2c). Taken together, these data indicate that macrophage cells regulate the pace of fracture repair. In old mice, young cells expressing F4/80 rejuvenate the repair process and aging of the F4/80 cell population will delay healing in the young skeleton.

### Young and old macrophages differentially secrete proteins

**Fig. 3:**
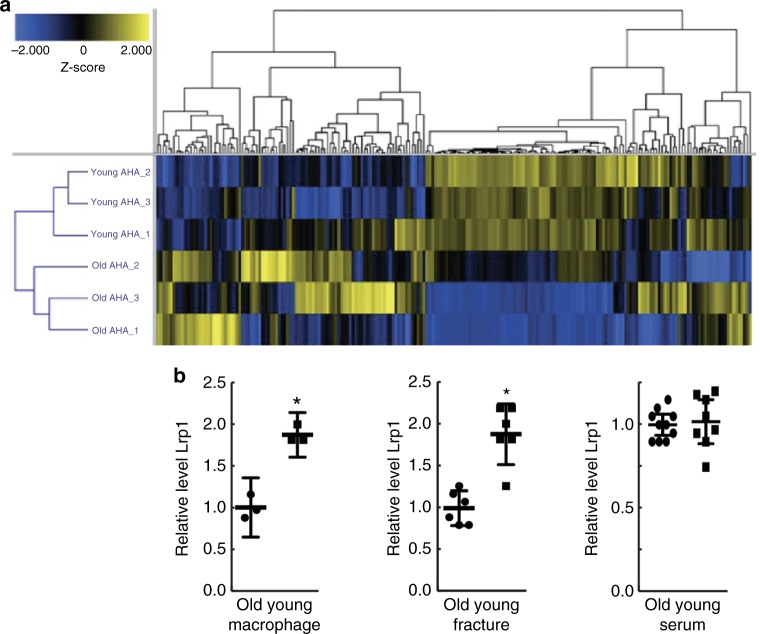
Differential protein secretion between young and old macrophage cells. **a** Heat map of the secreted proteins identified in the macrophage cultures from three old and three young mice, illustrating that there are differentially secreted proteins that segregate between old and young cells. **b** Elisa assay showing differential levels of Lrp-1 in old and young macrophages (*n* = 3); fracture callouses (*n* = 6) 1 week following fracture; or in the serum of young or old mice (*n* = 9 for old and 8 for young). Each data point is shown, along with the mean and 95% confidence interval for each experimental group. An asterisk indicates a significant difference of *p* < 0.05 compared to the control group, paired *t*-test (macrophage and callouses) and ANOVA (serum)

As we found that conditioned media from young cells increased osteogeneic differentiation, we investigated whether young and old bone marrow macrophages differentially secreted proteins that might influence osteogenesis. Macrophages derived from 3 young and 3 old C57BL/6J mice were established in methionine-free media containing azidohomoalanine (AHA)^18^. Conditioned media from these cultures were found to rejuvenate fracture repair to the same degree as cultures not labeled with AHA. The media were subjected to a click-chemistry reaction to enrich for secreted proteins and peptides, and AHA-incorporated peptides were subjected to mass spectrometry analysis. In unsupervised clustering analysis, the 6 secretomes surrogated into two groups with the secretomes from young cells clustering together and the secretomes from old cells clustering together. Using a 1% false discovery rate and a 1.5-fold change difference as cutoffs, we identified 56 proteins that were more abundantly expressed in young than old macrophage cultures and 6 proteins that were more abundant in old than young macrophages (Fig. 3 and Supplementary Table 1). The raw data are deposited in the MassIVE database (accession number, MSV000083009).

To determine whether differentially secreted factors might alter osteogenesis in old mice, we investigated one of the proteins that is produced at high levels in young macrophage cells. The mass spectrometry sequence of one of the most upregulated proteins in young macrophage cells mapped to soluble low-density lipoprotein receptor-related protein one (Lrp1). We selected this protein, as it is known to play a role in bone biology^19^ and it might alter β-catenin signaling, as β-catenin is known to play a role in impaired fracture healing with aging^9^. Lrp1 was the most differentially secreted protein that net these criteria. Lrp1 is in the same family as other co-receptor for Wnts. However, although it is not a typical receptor^20,21^, it can function as a Wnt anatogonist^21^. Soluble Lrp1 (sLrp1) is an evolutionarily conserved portion of the protein that can be released from the cell surface by transmembrane metalloproteinases or other processing, resulting a protein that can be detectable in plasma^22–24^. It is also known to be secreted in injury, such as to a nerve^25^, and in systemic inflammation^26^. Analyzing young and old macrophage conditioned media using ELISA, we verified an increase in the abundance of the sLrp1 in young macrophage conditioned media (Fig. 3b). The protein level was also increased in fractures from young mice compared to that in old mice, but not in the serum of these mice. This is consistent with the notion that sLRP1 being liberated from macrophage cells at the repair site, rather than being generally increased as the animals age (Fig. 3b).

### Lrp1 plays a role in the rejuvenation of fracture repair

**Fig. 4:**
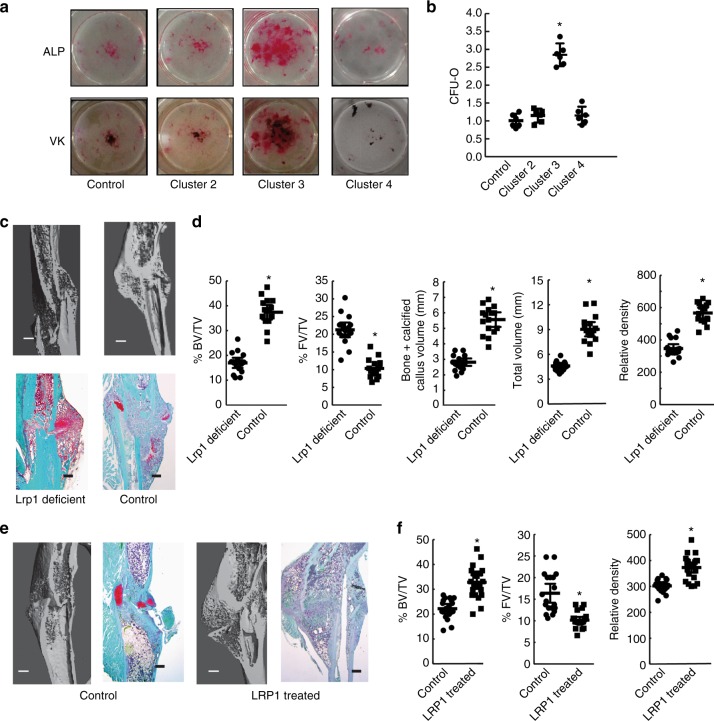
Low-density lipoprotein receptor-related protein one in young macrophage cells plays a role in the rejuvenation of fracture repair. **a** and **b** CFU-O (Von Kossa (VK) or Alk Phosphate (ALP) staining) that form from bone marrow from old mice when treated with carrier (control) or various domains (clusters) of Lrp1. **a** Representative cell culture plates, and **b** each data point is shown as well as means and 95% confidence intervals for data for each experimental group, with the mean from control cultures defined as “1” (right) are shown, *n* = 6, comparison using ANOVA. **c** and **d** Bone marrow from young mice lacking *Lrp1* in macrophages or controls were transplanted into old mice, *n* = 9 males and 8 females for Lrp1-deficient mice and 8 males and 8 females for controls. **c** Representative micro-CT data and Safranin O stained histologic images 4 weeks after the fracture and **d** Graphs show bone volume/total volume (BV/TV) in %; total fibrous tissue/total volume (FV/TV) in %; bone plus calcified callous volume (mm^3^), total callous volume (mm^3^), or relative density. Each data point is shown as well as means and 95% confidence intervals for data for each experimental group. An asterisk over a data point shows a significance *p* < 0.05 compared to controls, comparison using ANOVA. **e** and **f** Treatment with recombinant Lrp1 cluster three by daily injections at the fracture site for 2 days following generation of a tibia fracture improves fracture repair in old mice. **e** Representative micro-CT data and Safranin O stained histologic images 4 weeks after the fracture. **f** Bone volume/total volume (BV/TV) in %; total fibrous tissue/total volume (FV/TV), or relative density. *n* = 12 males and 11 females for Lrp1 treatment and 10 males and 9 females for controls. Each data point is shown as well as means and 95% confidence intervals for data for each experimental group. An asterisk over a data point shows a significance *p* < 0.05 compared to controls, comparison using ANOVA

Lrp1 protein can be divided into domains, termed clusters^27^. Recombinant proteins to each domain were used to determine their ability to influence osteogenic differentiation in bone marrow cells. Old bone marrow stromal cells cultured in the presence of 20 nM of cluster 3 showed increased osteoblast differentiation and matrix mineralization, albeit to a lower level than in the presence of young macrophage conditioned media (Fig. 4a, b). To determine whether Lrp1 in macrophages is involved in the rejuvenation of fracture repair in old mice in vivo, we generated a mouse in which *Lrp1* can be deleted from monocyte/macrophage lineage cells under control of the lysozyme promoter (*LysM-cre*;*lrp1*^*f/f*^), as *LyzM* is expressed in myeloid lineage cells^28^. Old mice were transplanted with bone marrow from either young *LysM-cre*;*Lrp1*^*f/f*^ mice or young *Lrp1*^*f/f*^ mice. Macrophage-specific deletion of *Lrp1* substantially decreased the rescue effect of young hematopoietic cells on fracture repair in old mice 2 and 4 weeks following a fracture (Fig. 4c, d and supplemental data (Supplementary Fig. 5A). There were equal numbers of F4/80 stained cells and osteoclasts in both experimental groups (supplementary Fig. 5A–C). To determine whether treatment with sLrp1 could improve the pace of fracture repair in old mice, fractures in old mice were treated with two injections of recombinant Lrp1 cluster 3 or carrier. Treatment with recombinant Lrp1 increased the proportion of bone present, and the relative bone density at the fracture site, albeit not the degree observed when old mice were transplanted with young macrophage cells (Fig. 4e, f). Systemic administration of the same dose of LRP1, either via IP injection or via tail vein injection had no effect on fracture repair.

**Fig. 5:**
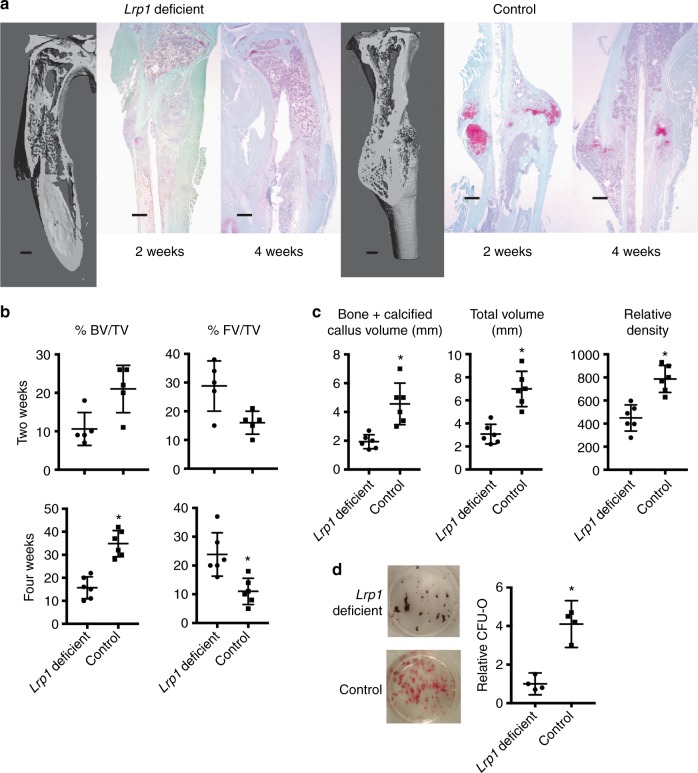
Fracture healing in young mice lacking *Lrp1* in macrophage cells. Fractures in *LysM-cre*;*Lrp1*^*f/f*^ mice were compared to *Lrp1*^*f/f*^ control mice, showing that young mice lacking Lrp1 in macrophages have a fracture repair phenotype reminiscent of that seen in wild-type old mice. **a** Representative micro-CT images at 4 weeks following the fracture, and safranin O staining histologic sections 2 and 4 weeks following fracture. The size marker is 0.5 mm. **b** Histomorphometric data as bone volume/total volume (BV/TV) in % and total fibrous tissue/total volume (FV/TV) in %. Each data point for each mouse analyzed is shown along with the mean and 95% confidence interval for each experimental group. An asterisk indicates a significant difference compared to the control group (*p* < 0.05). **c** Micro-CT data given as bone plus calcified callus volume (mm^3^), total callous volume (mm^3^), or relative density. Each data point for each mouse analyzed is shown along with the mean and 95% confidence interval for each experimental group. An asterisk indicates a significant difference (*p* < 0.05) compared to the control group. **d** Conditioned media from macrophages from *LysM-cre*;*Lrp1*^*f/f*^ mice or *Lrp1*^*f/f*^ mice were used in CFU-O differentiation. Conditioned media from macrophages lacking *Lrp1* resulted in fewer CFU-Os than conditioned media from control macrophages. Each data point is shown along with the mean and 95% confidence interval for each experimental group. An asterisk indicates a significant difference (*p* < 0.05) compared to the control group. Comparison using ANOVA in (**b**) and (**c**), and paired *t*-test in (**d**)

Although this data examined the role of Lrp1 in the rejuvenation of fracture repair, it does not identify its role in fracture repair in general. Interestingly, single nucleotide polymorphisms in the *LRP1* gene coding sequence are associated with low bone mass^29^. More recent studies show that Lrp1 regulates osteoblast proliferation and osteoblast-mediated osteoclastogenesis^30^, and Lrp1 deficiency in osteoclasts decreases bone mass^19^. Whereas Lrp1 is known to play a role in bone homeostats, in particular in osteoblasts and osteoclasts in remodeling, its function in macrophage cells in bone repair is unknown. We thus examined fracture repair in the young mice lacking *lrp1* in monocyte/macrophage lineage cells, to determine whether this would result in a fracture repair phenotype similar to that observed in old mice. There were no differences in baseline bone density between young *LysM-cre*;*Lrp1*^*f/f*^ mice or *Lrp1*^*f/f*^ mice (the mice used as donors in the rejuvenation studies). However, there was reduced osteogenic differentiation, and reduced bone formation during fracture repair in these mice (Fig. 5). This is a fracture repair phenotype reminiscent of that observed in old mice. Thus, although Lrp1 plays a role in bone homeostasis in a number of cell types, during fracture repair, Lrp1 in macrophage cells also plays a role in osteogenesis and the amount of bone at the fracture repair site.

One reason we selected Lrp1 is that in might regulate β-catenin-mediated signalling. Data on fracture repair and osteoblastic differentiation form undifferentiated mesenchymal cells shows that β-catenin plays different roles in different phases of the repair or differentiation process. At the initial phases of fracture repair, and for undifferentiated cells to become osteochondral progenitors, β-catenin needs to be precisely regulated^31^. Levels that are too high or too low will prevent differentiation to osteochondral progenitor cells, and instead result in cells adopting a fibroblastic like phenotype^33^. At a fracture site, this can cause a delayed or non-union. With aging, β-catenin levels are high in the initial phase of repair, and lowering the level could improve the quality of fracture healing^9^. We found that Lrp1 will decrease β-catenin levels in healing fracture in old animals (Supplementary Fig. 6). To determine whether β-catenin modulation is required for the effect of Lrp1 on repair in old animals, we used mice in which we could conditionally depleted β-catenin^32^. An adenovirus expressing Cre-recombinase was used to deplete β-catenin at the fracture site as in our, and other’s previous work showing this effectively depletes loxP flanked sequences at the site of bone repair^16,33–35^. When we depleted β-catenin at the fracture site in old mice, Lrp1 treatment no longer had an effect on fracture repair. In addition, Lrp1 did not alter CFU-O numbers in marrow cells from β-catenin-deficient mice (Supplementary Fig. 6).

## Discussion

Here we show that macrophage cells from young animals can rejuvenate fracture repair in old animals. This rejuvenation is associated with the factors produced by young macrophage cells that enhance osteogenic differentiation of bone marrow stromal cells. Indeed, depletion of one such factor from macrophage cells partially abrogated the rejuvenation effect, and treatment with this factor alone partially rejuvenated the fracture repair phenotype in old mice. Whereas other studies show that sharing the circulation of a young animal can rejuvenate repair^36–38^, our data provide evidence that macrophage cells and factors that derive from these cells can rejuvenate repair. Importantly, this data also show that the macrophage phenotype orchestrates the bone repair process, as the fracture phenotype in our transplant studies mirrors that of the donor animal, not the host.

We used both bone marrow transplantation and parabiosis experiments to study rejuvenation. Whereas there are physiologic stresses associated with parabiosis, this model maintains the young hematopoietic progenitors and monocyte/macrophage cells in a young niche, eliminating changes in monocyte lineage cell due to their environment. In contrast, bone marrow transplantation studies have the disadvantage that cells may lose the phenotype from the donor niche, but lack the physiologic stresses associated with parabiosis. New cells from hematopoietic progenitors will eventually replace transplanted F4/80-positive cells. As the effect of macrophage cells on fracture repair occurs within the initial phase of fracture repair, a time when undifferentiated mesenchymal cells become osteochondral progenitors, the effect of young or old F4/80-positive cells in the repair process can be assayed using the fractionated bone marrow transplantation approach. Showing similar effects using these two approaches provides evidence that the changes we observed are due to the effect of macrophage cells in the repair process, rather than related to changes associated with the specific models utilized.

Although Lrp1 has not previously been implicated in fracture repair, it is known to play a role in bone homoeostasis, osteoblast and osteoclast function, and macrophage function. Mice lacking Lrp1 in macrophages have more extensive atherosclerosis when crossed into an LDL receptor-deficient mouse. A similar phenotype is observed when bone marrow from Lrp1-deficient mice are transplanted into LDL receptor-deficient mice^39,40^. Macrophage cells lacking Lrp1 also are defective in their ability to clear certain proteins, such as von Willebrand factor, and the production of factors such as TGF-β^41,42^. Lrp1 plays a role in the regulation of bone remodeling. In humans, a single nucleotide polymorphism in *LRP1* is linked to low bone density^29^. Mice in which osteoblasts lack *Lrp1* show osteoporosis due to highly increased osteoclast numbers and bone resorption^30^. In addition, mice lacking *Lrp1* in osteoclasts show a dramatically decreased trabecular bone mass with substantially more osteoclasts^19^. Here we extend the repertoire of cells in which *Lrp1* plays a role in bone to include macrophages. *Lrp1* in macrophage cells primarily plays a role in bone in repair, as mice lacking *Lrp1* in these cells do not demonstrate an obvious bone phenotype baseline. Systemic Lrp1 administration had little effect of healing, perhaps due to higher local levels when injected into the healing fracture. Lrp1 is unlikely to be the only factor important in how macrophage cells regulate bone repair, and in rejuvenation. Indeed, treatment with Lrp1 has less of an effect on rejuvenation than young macrophage cells. Taken together, our data show that local macrophage cells produce factors that can rejuvenate fracture repair, and that Lrp1 is but one such factor.

Many cell types increase protein secretion with senescence^43–45^, a process associated with aging, as well as some repair processes^14,46^. Whereas we found some proteins secreted at higher levels in older macrophages, interestingly there were far more proteins secreted at higher levels in young cells. Whereas there is little information available on the effect of aging on macrophage cell secretion, a few studies examining secretion of individual proteins with age, also found lower levels of protein secretion of as cells age^14,46^. Many of the proteins we found increased in young cells were lysosomal and cell surface proteins. One possibility is that lysosomal regulation and autophagolysosomal system^47^ are more effectively activated in the young cells, as secretory autophagy^48^ can regulate protein secretion in macrophages^49^. As macrophages produce have less proteins with aging, this could be one mechanism by which younger macrophages stimulate improved regeneration, as the additional factors may produce a milieu for other cells to adopt a more favorable phenotype for repair. Other factors differentially secreted by macrophages identified in our study could also be related to Lrp1. One such protein which is secreted at higher levels in old cells is PAI-1, which can also regulate β-catenin^50^. PAI-1 is also known to be differentially regulated in aging, and is associated with longevity^51^. Although lysosomal regulation, increased protein secretion, and a relationship with other factors such as PAI-1 is only speculative, this is consistent with the notion that not one, but a variety of differentially related factors are responsible for the rejuvenation effect. Whereas it is possible that other effects of Lrp1 deficiency on macrophages function might cause the observed phenotype, the finding that Lrp1 protein stimulates osteogenesis, suggests that Lrp1 secretion plays a role in the rejuvenation of fracture repair.

There is increasing support for the concept that macrophage phenotypes are epigenetically regulated, influenced by the niche of their site of origin, well as the environment to which they migrate^52,53^. Our findings that macrophage cells retain the capacity to orchestrate fracture healing associated with the animal of origin rather than their transplanted host supports the notion that the niche of their site of origin is important in maintaining their phenotype. Indeed, these cells maintain the ability to rejuvenate fracture repair for an extended period of time after transplantation, where they initiate the repair processes, and achieve this through production of secreted factors. Thus, the macrophage phenotype plays a crucial role of in regulating the pace and quality of fracture repair.

Many factors and cells play a role in fracture repair. Our in vivo data uses a fracture repair model in which periosteal progenitors can be activated to differentiate into chondrocytes ultimately stimulating osteogenesis. In contrast, our in vitro studies show an effect on undifferentiated stromal cells becoming osteoblasts. Thus, macrophages and factors they produce can rejuvenate osteogenesis from mesenchymal cells from multiple sources.

A slower pace of repair and less osteogenic differentiation increases the chance that fracture go onto a delayed union or non-union, whose treatment is associated with decreased mobility even mortality in older individuals. As such, delayed fracture healing is a major health issue in aging, and strategies to improve the pace of repair and prevent non-union will substantially improve patient outcomes^54^. Whereas macrophages are known to play a role in repair and regeneration, there is scant evidence suggesting a role in the rejuvenation process^55^ and prior studies do not identify secreted factors responsible for the effect. Here we show that young macrophage cells and play a role in the rejuvenation process, suggesting a therapeutic approach to fracture rejuvenation.

## Methods

### Mice

All of the animals in this study were C57BL mice. We obtained institutional ethical approval through Duke University’s Office of Animal Welfare Assurance for all of the animal experiments (IACUC). We used equal numbers of male and female mice in all of the studies, except for parabiosis studies in which only female mice were utilized. As we were studying age differences, mice of various ages were investigated, and the specific ages are outlines in each study. To effectively target macrophage lineage cells in vivo, we used macrophage Fas-induced apoptosis transgenic mice (MaFIA). This mouse expresses a mutant human FK506 binding protein 1A, 12 kDa (FKBP12) driven by the mouse *Csf1r*, (colony stimulating factor 1 receptor-expressed almost uniquely by macrophages) promoter. Expression is limited to macrophages, and when the mice are treated with the dimerizing reagent, AP20187, this induces apoptosis in macrophages. AP20187 was dissolved in 100% ethanol to a concentration of 13.75 mg/ml and stored at −20 °C. This stock solution was further diluted to a 0.55 mg/ml in an injection solution of 45 ethanol, 10% PEG-400, and 1.7% Tween in water. Tail vein injections of AP20187 at a dose of 10 mg per kg mouse were used to ablate macrophages. Macrophages from the MaFIA mice also express EGFP. Five days of treatment effectively depletes 90% of macrophage cells^15,56–58^, a finding we confirmed in our mice using flow cytometry for GFP-positive cells in blood samples (Supplementary data Fig S1). To delete, *Lrp1* we used a conditional knockout mouse, by crossing a mouse expressing floxed null alleles for Lrp1^59^ with a mouse in which cre is driven by *LysM*^28^. We previously showed that *LysM-cre* effectively targets macrophage lineage cells in repair processes in mice^11,60^. To determine the contribution of β-catenin to the fracture phenotype in old mice, we examined animals in which we can conditionally knockdown β-catenin. Mice expressing ether homozygous *Catnb*^*tm2Kem*^ alleles were used^32^. When treated with Cre-recombinase, this results in a β-catenin-null allele. An adenovirus expressing Cre-recombinase (Ad-Cre, Vector biolabs) was used to drive recombination in the fracture site in mice. As a control, the same virus expressing, but GFP was used. 10^9^ pfu of virus was injected into the fracture site. Data from our group and others show that this effectively drives recombination in >80% of cells at the fracture site^33–35^. Half the mice were treated with Ad-Cre and the other half with Ad-GFP as a control. Western analysis was used to confirm depletion of β-catenin.

### Western analysis

Protein extracts were separated by electrophoresis on a polyacrylamide gel and transferred onto a nitrocellulose membrane. The membranes were probed with a primary antibody that was detected using a secondary antibody and ECL reagents (Amersham, Piscataway). To determine the level of β-catenin, using an anti-β-catenin antibody (from BD Biosceinces, Clone 14, catalogue number 610153) at a dilution of 1:1000. As a loading control, an anti GAPDH antibody at a dilution of 1:5000 was used (Abcam, 6C5, catalogue number ab8245) on a reprobed membrane. Densitometry comparing β-catenin to GAPDH intensity was used to determine relative β-catenin protein level between samples.

### Parabiosis

We investigated young mice, 4 months old, equivalent of young adult (25 years old), or old mice, 20 months of age, equivalent of humans in their late 60’s. Young or old MaFIA mice were anastomosed to old C57BL/6 mice. We deleted macrophage cells from young MaFIA mice and examined healing of a tibia fracture^16,35^. Half the paired mice were treated with 10 mg/kg AP20187 and the other half were treated with the vehicle injection solution, starting 4 weeks following parabiosis, a time in which mice will share blood flow. AP20187 or vehicle was administered daily for 5 days starting 1 day prior to fracture surgery. Select mice were analyzed for GFP expressing cells (expressed by macrophages in MAFIA mice) at the time of sacrifice verifying effective deletion of macrophages. Bone marrow cells were treated with red blood cell lysis buffer, counted and resuspended at 1 × 10^7^ cells in 1X PBS/1% FBS. Cells were incubated with propidium iodide to stain for dead cells. A MoFlow Astrios flow cytosmear (Beckman) was utilized. Forward scatter and side scatter was set with linear scale and voltage adjusted to include most of the cells. The 488 nm blue laser was used to detect GFP. A sample negative for GFP was used as a control to determine the gating strategy. Based on the spread of the cells in the negative control, the GFP gate was drawn that do not include any cells in the negative control. Flowjo software was used. (Supplementary Fig. 1). One week later, a fracture was generated in one tibia. Only female mice were studied for the parabiosis studies, and fractures were examined after 2 weeks as per the requirements of our local animal care committee (IACUC). We studied 12 animals in each group based on the means and standard deviation from our previous publications on fracture healing using the same method and analysis^9,33,61^, selecting a number that would have the power to detect a 20% difference in bone volume/total volume, but some animal pairs suffered complications, and were not able to be maintained until the planned endpoint. The numbers of animals in each comparison are in the figure legend.

### Macrophage cell cultures and cell sorting

Bone marrow cells were plated at a density of 3.9 × 10^5^ cells per well of 12-well plates in AMEM media, 10% FBS, and 40 ng/ml recombinant M-CSF, which was replaced after 3 days^62^. For conditioned media experiments, the bone marrow cells were cultured with 40 ng/ml recombinant M-CSF, and after 3 days, media were collected, mixed 1:1 with fresh osteoblast differentiation media (see colony forming unit osteoblastic methods below), and used to culture bone marrow stromal cells for use with the colony unit osteoblastic assay. To isolate macrophage cells, bone marrow cells were treated with red blood cell lysis buffer, counted and resuspended at 1 × 10^7^ cells in 1X PBS/1% FBS. Cells were stained with Alexa647-conjugated F4/80 antibody diluted 1:150 (BioLegend, catalogue number 12312) incubated with propidium iodide to stain for dead cells, and sorted using a MoFlow Astrios cell sorter (Beckman).

### Bone marrow transplantation

We undertook bone marrow transplantation experiments to determine whether macrophage cells alone from young animals can rescue the fracture repair phenotype in old animals. Bone marrow cells were collected from MAFIA mice sorted into separate F4/80-positive (F+) and negative-(F−) fractions from old and young mice as detailed in the macrophage cell cultures and cell sorting Methods section. We then combined cells from the fractious (reconstituted at a F+ to F− ratio of 1:9) to produce bone marrow that is F− old and F+ young; F+ young, and F− old; F+ and F− old; and F+ and F− young bone marrow cells. These were used in bone marrow transplant experiments as in our previous work^9^. Mice were irradiation: 900 cGy of gamma radiation and fractured 2 months after transplantation using 1 × 10^6^ cells in 200 μl PBS via tail vein injection. In select mice, the number of fluorescent expressing macrophage cells per high powered field were determined to verify macrophage engraftment from the recipient mice (Supplementary Fig. 3). We studied 10 male and 10 female animals in each group, but some suffered complications, and were not able to be maintained until the planned endpoint. Our previous studies showed little difference between fracture phenotypes between male and female old mice^9^. The numbers of animals in each comparison are in the figure legend.

### Fracture generation and histologic healing analysis

An open tibia fracture was generated by surgically exposing the proximal 1/3 tibia, and cutting the bone using an osteotome. The fracture was stabilized this with an intramedullary rod, so the mice had little discomfort and could bear weight while healing^16,34^. Mice were killed and healing fractures analyzed. For histology, collected fractures were fixed in 10% formalin overnight at 4 °C, decalcified in 14% EDTA (pH 7.2) for 2 weeks and paraffin embedded. Serial sections of 5 μm were deparaffinized and rehydrated to water for hematoxylin and eosin (H&E), Safranin O, and tartrate-resistant acid phosphatase (TRAP) staining.

### Immunohistochemistry and histomorphometry

For F4/80 immunohistochemistry, antigen retrieval was performed with 20 μg/ml of proteinase K for 10 min and stained with primary rat- anti-mouse F4/80 (AbD Serotec, catalogue number MCA497) antibody diluted 1:100. To detect GFP expression, sections were deparaffinized and incubated in citric acid-based antigen unmasking solution (VECTOR) at 80 °C for 15 min. After blocking with 10% goat normal serum, the sections were incubated with anti-GFP antibody (Cell signaling, 1:200) for 2 h at room temperature. Immune complexes were detected using secondary antibodies conjugated to Alexa Fluor 594 goat anti-rabbit IgG (Invitrogen, 1:2000). Hoechst (Thermo Fisher, 1:2000) was used to detect nuclei. Images were acquired on Axio Imager Z2 upright microscope (Zeiss). Antibodies used and catalogue numbers were: GFP (D5.1) XP® Rabbit mAb #2956 Cell signaling; Alexa fluor 594 goat anti-rabbit A11037 Invitrogen; and Hoechst 33342 #62249 Thermo Fisher for nuclear counterstaining. Computer assisted histomorphometry was used to analyze the type and proportion of cells types and tissue types present in the callous using Safranin-O stained sections (to identify bone, cartilage, and fibrous tissue). For histomorphometry, 12 sections were used to determine bone and callus parameters. The fracture callous was defined as 2 mm on each side of the fracture line. Osteoclasts were quantified as the surface of osteoclasts to total bone surface. To quantify the abundance of F4/80-positive cells, the percentages of F4/80-positive cells to total cells were determined.

### Recombinant protein treatment

For LRP-1 treatment studies, 5 μg of recombinant LRP1 cluster three (Recombinant Human LRP-1 Cluster III from R and D systems, Catalog Number: 4824-L3) or a control protein carrier as control were injected using a 27 gauge needle at the fracture site after the generation of the fracture and again the next day. The dose was selected from prior studies analyzing local LRP-1 treatment in neural tissues^25,63^.

### Micro-CT analysis

Assessment using a micro-CT was performed using techniques as in our previous work to determine calcification at the fracture callous^61,64^. For parabiosis and studies of bone marrow transplantations using F4/80 positive and negative cell fractions, the Skyscan 1174 (Skyscan, Kontich, Belgium) with image acquisition performed at 50 kV and 800 µA. For studies of Lrp1-deficient cell transplants and Lrp1 recombinant proetin treatment, the VivaCT 80, Scanco, Brüttisellen, Switzerland at 55 kVp and 145 µA was utilized. A hydroxyapatite (HA) calibration phantom was used to scale values of linear attenuation for the calcified tissues to bone density values (mg HA/cm^3^) using both devices. Calcified tissues were segmented from soft tissues using a global thresholding procedure with a threshold of 480 mg HA/cm^3^, which represents 45% of the attenuation of mature cortical bone. Morphometric parameters were quantified using a direct 3-D approach at the site of fracture that included 2 mm proximal and distal from the fracture location. Measurements in this volume of interest included the total volume (TV in mm^3^), volume of calcified callus (BV in mm^3^), and regional mean relative bone mineral density. Statistical analysis was performed using a two-way analysis of variance (ANOVA) and a Tukey post hoc analysis with data reported at 95% confidence level. Because, we studied genetically identical animals, we did not use a formal randomization process. All analyses were done by an observer blindered to the experimental groups.

### Bone marrow stromal cell cultures and CFU-O assay

Bone marrow stromal cells were obtained from aspirates of the from tibiae of unfractured mice. Stromal cells were selected based on their adherence to tissue culture plastic. Aspirated cells were plated (AMEM and 10% FBS) for 7 days. A total of 3.9 × 10^6^ cells were plated per well. To induce osteogenic differentiation, the media were changed to osteogenic media (plating media with 30 μM ascorbic acid, 8 mM phosphate, and 10 nM dexamethasone). Media were changed every other day thereafter. After 15 days in differentiation media, cells were stained with alkaline phosphatase (ALP, indicates osteoblasts), or von kossa (VK, indicates mineral formation). Colony-forming units (CFUs) were defined as individual clusters of at least 30 cells.

For conditioned media experiments, media were transferred directly from well to well after 2 days of conditioning. Media were collected, mixed 1:1 with fresh osteoblast differentiation media, and used to culture bone marrow stromal cells. In each case control conditioned media from a control was used as a comparison. Conditioned media were obtained from additional bone marrow stromal cultures or from macrophage cultures.

### Identification of differentially secreted proteins

Quantitative two-dimensional liquid chromatography–tandem mass spectrometry (LC/LC–MS/MS) was used to detect differentially secreted proteins between young and old macrophages in cell culture. To be able to more easily identify secreted proteins from the relatively small cell numbers of murine macrophages in culture, we used azidohomoalaine (AHA) pulse chase labeling^65^. The dose and duration of AHA was optimized, and we verified that the AHA-labeled conditioned media from the young macrophage cells would be able to rescue CFU-O numbers in old bone marrow cells. Equal number of macrophage cells were cultured as described above for 24 h in media devoid of methionine with 40 ng/ml M-CSF, containing 100 μM of AHA.

For each aliquot of media, 2 × 10 ml were precipitated by addition of 2.5 vol of ice cold acetone, followed by incubation at −20 °C for 30 min. Precipitated protein was collected by centrifugation. Supernatants were decanted, and pellets were washed at room temp with 70% acetone. Pellets were resuspended in ~2 ml of 100 mM Tris, pH 8, 1% SDS (4 ml total per sample) using probe sonication. Proteins were further denatured by heating at 85 °C for 10 min followed by alkylation with 10 mM iodoacetamide (IAM) for 30 min in the dark. Samples were stored frozen at −80 °C.

Twenty milligrams of each sample was adjusted to 2 ml with Tris/1% SDS and tumbled with 150 µl of DBCO-agarose slurry (Click Chemistry Tools) overnight at room temperature. Following overnight incubation, resins were transferred to Bio-Rad Micro Biospin columns and washed by gravity with 3 × 1 ml each of: Tris/1%SDS, Tris/1%SDS/10 mM DTT; H_2_O; 8 M Urea; H_2_O; 10X PBS; H_2_O; 20% Isopropanol; and 20% acetonitrile (MeCN). Following the last organic wash, beads were swelled in 50 mM ammonium bicarbonate (AmBic) at 4 °C for 2 h and transferred to 1.5 ml microcentrifuge tubes. Following centrifugation at 1000×*g* for 30 s, and removal of supernatant, beads were reduced with 100 µl of 50 mM ammonium bicarbonate containing 20 mM DTT and 0.2% acid-labile surfactant (ALS-1) was added to give a final volume of 200 µl with 0.1% ALS-1 and 10 mM DTT. Resins were heated at 80 °C for 10 min followed by addition of 25 mM IAM and shaken at room temp for 30 min. Resins were washed 2× with 500 µl of 0.1% ALS-1. After the last centrifugation, remaining liquid was removed with a 28 gauge needle. Finally, 125 µl of 50 mM AmBic containing 0.1% ALS-1 and 1 µg Sequencing Grade Modified Trypsin (Promega) were added to each sample. Tubes were incubated with shaking at 950 rpm at 37 °C overnight. After trypsin digestion, supernatants were recovered and beads washed 2× with 125 µl of 50 mM AmBic. After combining eluents and washes, samples were acidified for 2 h at 60 °C to hydrolize the ALS-1, centrifuged, and supernatants were frozen at −80 °C.

Peptides were lyophilized and resuspended in 20 µl of 2% MeCN/200 mM ammonium formate (pH 10). Pools were generated by mixing equal amounts of the peptides recovered from Met-labeled old or young macrophages, and an additional QC pool was generated by mixing equal volumes of peptide digests recovered from all six AHA-labeled samples. In this way, peptides were standardized between samples by the amount of protein excreted per period of time by the same number of cells. A total of 25 fmol ADH1_YEAST MassPrep Standard (Waters) was added per µl of reconstituted peptide digest.

Quantitative two-dimensional liquid chromatography–tandem mass spectrometry (LC/LC–MS/MS) was performed on 2.5 µl (3 µg) using two-dimensional liquid chromatography in a high–low pH reversed phase/reversed phase configuration on a nanoAcquity UPLC system (Waters) coupled to a Synapt G2 HDMS high resolution accurate mass tandem mass spectrometer (Waters) with nanoelectrospray ionization. Peptides were first trapped at 2 µl/min at 97/3 v/v water/MeCN in 20 mM ammonium formate (pH 10) on a 5 µm XBridge BEH130 C18 300 µm × 50 mm column (Waters). A series of step-elutions of MeCN at 2 µl/min was used to elute peptides from the 1st dimension column. Ten steps of 7.4%, 10.8%, 12.6%, 14.0%, 15.3%, 16.7%, 18.3%, 20.4%, 23.5%, and 50% MeCN were utilized; these percentages were optimized for delivery of an approximately equal load to the 2nd dimension column for each fraction. For 2nd dimension separation, the eluent from the 1st dimension was first diluted tenfold online with 99.8/0.1/0.1 v/v/v water/MeCN/formic acid, respectively, and trapped on a 5 µm Symmetry C18 180 µm × 20 mm trapping column (Waters). The 2nd dimension separations were performed on a 1.7 µm Acquity BEH130 C18 75 µm × 150 mm column (Waters) using a linear gradient of 7–35% MeCN with 0.1% formic acid over 37 min, at a flow rate of 0.5 µl/min and column temperature of 35 °C. Data collection on the Synapt G2 mass spectrometer was performed in either ion-mobility enabled data-independent acquisition (HD-MSE) mode to generate qualitative and quantitative data, using 0.6 s alternating cycle time between low (6 V) and high (27–50 V) collision energy (CE) or data-dependent acquisition (DDA) to generate qualitative only data, using a 0.6 s precursor MS scan followed by three MS/MS scans of the highest abundant precursor ions. A 60 s exclusion limit was employed for DDA acquisitions. Scans performed at low CE measure peptide accurate mass and intensity (abundance), whereas scans at elevated CE allow for qualitative identification of the resulting peptide fragments via database searching. The total analysis cycle time for each sample injection was ~10 h.

Samples were then analyzed as follows. An initial conditioning run, which also served to confirm optimal sample loading, was performed using the QC pool containing the mixture peptides enriched from all of the AHA-labeled cell culture media. Next, the two Met-labeled pools were analyzed, followed by QC1, Old-AHA-1, Old-AHA-2, Young-AHA-2, QC2, Young-AHA-3, Young-AHA-1, Old-AHA-3, and QC3, all using HD-MSE acquisition. In order to improve peptide/protein identifications, two additional analyses, of a QC pool and Young-AHA-1, were performed using DDA mode. Following the 14 analyses, all data, except the initial conditioning run, was imported into Rosetta Elucidator v3.3 (Rosetta Biosoftware, Inc), and all LC/LC–MS/MS runs were aligned based on the accurate mass and retention time of detected ions (“features”) using PeakTeller algorithm (Elucidator). MS/MS data were searched against an NCBI RefSeq database (*mus musculus* taxonomy), which also contained bovine serum albumin, a reversed-sequence “decoy” database for false-positive rate determination as well as yeast ADH1, which was added to samples as a surrogate internal standard. Database searching of HD-MSE was performed with Protein Lynx Global Server (v2.5.2, Waters Corp) with trypsin specificity and up to 2 missed cleavages, fixed modification of Cys (carbamidomethyl), variable modification of N and Q (deamidation,+1 Da) and M (oxidation,+16 Da). DDA data were searched using Mascot v2.4 within Rosetta Elucidator using semitrypsin specificity, and including the additional of variable modification on Pro (hydroxyl). Data were annotated at a <1% peptide false discovery rate using the PeptideProphet algorithm within Elucidator. For quantitative processing, the data were first curated to contain only high quality peptides with appropriate chromatographic peak shape and the dataset was intensity scaled after removal keratin contaminants; the final quantitative dataset contained 1065 peptides, which mapped to 210 proteins.

### Elisa

Differential expression of Lrp1 in tissue or serum was measured using an Elisa analysis according to the manufacturer’s instructions (Aviva Systems Biology, San Diego, CA, murine LRP1 ELISA Kit, OKEH01469). Protein was extracted from tissue following two freeze-thaw cycles and centrifugation of homogenates for 5 min at 5000×*g*, 4 °C. Protein was analyzed from serum after centrifugation for 15 min at 1000×*g* at 4 °C.

### Statistical analysis

Statistical analysis was conducted using GraphPad Prism v5 and Microsoft Excel 2011 statistical software. Paired *t*-tests or ANOVA were conducted depending on the comparison groups and results are reported as mean + 95% confidence interval. Results were deemed significant when *p* < 0.05. The sample sizes were estimated based on previous experiments. As we studied genetically identical animals, we did not use a formal randomization process.

## Electronic supplementary material

Supplementary Information

## Data Availability

The authors declare that all data supporting the findings of this study are available within the article and its Supplementary Information Files or from the corresponding author upon reasonable request. Raw data for the secretome have been deposited in the MassIVE database under accession code MSV000083009.

## References

[1] Tonna EA (1964). Fracture callus formation in young and old mice observed with polarized light microscopy. Anat. Rec..

[2] Xing Z (2010). Multiple roles for CCR2 during fracture healing. Dis. Model Mech..

[3] Abdallah BM, Haack-Sorensen M, Fink T, Kassem M (2006). Inhibition of osteoblast differentiation but not adipocyte differentiation of mesenchymal stem cells by sera obtained from aged females. Bone.

[4] Yadav VK (2009). A serotonin-dependent mechanism explains the leptin regulation of bone mass, appetite, and energy expenditure. Cell.

[5] Strube P (2008). Influence of age and mechanical stability on bone defect healing: age reverses mechanical effects. Bone.

[6] Meyer RA (2001). Age and ovariectomy impair both the normalization of mechanical properties and the accretion of mineral by the fracture callus in rats. J. Orthop. Res..

[7] Clement ND, Aitken SA, Duckworth AD, McQueen MM, Court-Brown CM (2011). The outcome of fractures in very elderly patients. J. Bone Joint Surg. Br..

[8] Calori GM, Albisetti W, Agus A, Iori S, Tagliabue L (2007). Risk factors contributing to fracture non-unions. Injury.

[9] Baht GS (2015). Exposure to a youthful circulaton rejuvenates bone repair through modulation of beta-catenin. Nat. Commun..

[10] Wynn TA, Vannella KM (2016). Macrophages in tissue repair, regeneration, and fibrosis. Immunity.

[11] Vi L (2015). Macrophages promote osteoblastic differentiation in-vivo: implications in fracture repair and bone homeostasis. J. Bone Miner. Res..

[12] Alexander KA (2017). Resting and injury-induced inflamed periosteum contain multiple macrophage subsets that are located at sites of bone growth and regeneration. Immunol. Cell Biol..

[13] Linehan E, Fitzgerald DC (2015). Ageing and the immune system: focus on macrophages. Eur. J. Microbiol. Immunol..

[14] Gibon E (2016). Aging affects bone marrow macrophage polarization: relevance to bone healing. Regen. Eng. Transl. Med..

[15] Burnett SH (2004). Conditional macrophage ablation in transgenic mice expressing a Fas-based suicide gene. J. Leukoc. Biol..

[16] Chen Y (2007). Beta-catenin signaling plays a disparate role in different phases of fracture repair: implications for therapy to improve bonehealing. PLoS Med..

[17] Ajami B, Bennett JL, Krieger C, McNagny KM, Rossi FM (2011). Infiltrating monocytes trigger EAE progression, but do not contribute to the resident microglia pool. Nat. Neurosci..

[18] Dieterich DC, Link AJ, Graumann J, Tirrell DA, Schuman EM (2006). Selective identification of newly synthesized proteins in mammalian cells using bioorthogonal noncanonical amino acid tagging (BONCAT). Proc. Natl Acad. Sci. USA.

[19] Lu D (2018). LRP1 suppresses bone resorption in mice by inhibiting the RANKL-stimulated NF-kappaB and p38 pathways during osteoclastogenesis. J. Bone Miner. Res..

[20] Terrand J (2009). LRP1 controls intracellular cholesterol storage and fatty acid synthesis through modulation of Wnt signaling. J. Biol. Chem..

[21] Zilberberg A, Yaniv A, Gazit A (2004). The low density lipoprotein receptor-1, LRP1, interacts with the human frizzled-1 (HFz1) and down-regulates the canonical Wnt signaling pathway. J. Biol. Chem..

[22] Quinn KA, Pye VJ, Dai YP, Chesterman CN, Owensby DA (1999). Characterization of the soluble form of the low density lipoprotein receptor-related protein (LRP). Exp. Cell Res..

[23] Grimsley PG, Quinn KA, Chesterman CN, Owensby DA (1999). Evolutionary conservation of circulating soluble low density lipoprotein receptor-related protein-like (“LRP-like”) molecules. Thromb. Res..

[24] Quinn KA (1997). Soluble low density lipoprotein receptor-related protein (LRP) circulates in human plasma. J. Biol. Chem..

[25] Gaultier A (2008). A shed form of LDL receptor-related protein-1 regulates peripheral nerve injury and neuropathic pain in rodents. J. Clin. Invest..

[26] Gorovoy M, Gaultier A, Campana WM, Firestein GS, Gonias SL (2010). Inflammatory mediators promote production of shed LRP1/CD91, which regulates cell signaling and cytokine expression by macrophages. J. Leukoc. Biol..

[27] Lillis AP, Van Duyn LB, Murphy-Ullrich JE, Strickland DK (2008). LDL receptor-related protein 1: unique tissue-specific functions revealed by selective gene knockout studies. Physiol. Rev..

[28] Clausen BE, Burkhardt C, Reith W, Renkawitz R, Forster I (1999). Conditional gene targeting in macrophages and granulocytes using LysMcre mice. Transgenic. Res..

[29] Sims AM (2008). Genetic analyses in a sample of individuals with high or low BMD shows association with multiple Wnt pathway genes. J. Bone Miner. Res..

[30] Bartelt A (2018). Lrp1 in osteoblasts controls osteoclast activity and protects against osteoporosis by limiting PDGF-RANKL signaling. Bone Res..

[31] Day TF, Guo X, Garrett-Beal L, Yang Y (2005). Wnt/beta-catenin signaling in mesenchymal progenitors controls osteoblast and chondrocyte differentiation during vertebrate skeletogenesis. Dev. Cell.

[32] Brault V (2001). Inactivation of the beta-catenin gene by Wnt1-Cre-mediated deletion results in dramatic brain malformation and failure of craniofacial development. Development.

[33] Chen Y (2007). Beta-catenin signaling pathway is crucial for bone morphogenetic protein 2 to induce new bone formation. J. Biol. Chem..

[34] El-Hoss J (2012). A murine model of neurofibromatosis type 1 tibial pseudarthrosis featuring proliferative fibrous tissue and osteoclast-like cells. J. Bone Miner. Res..

[35] Ghadakzadeh S, Kannu P, Whetstone H, Howard A, Alman BA (2016). beta-Catenin modulation in neurofibromatosis type 1 bone repair: therapeutic implications. FASEB J..

[36] Sinha M (2014). Restoring systemic GDF11 levels reverses age-related dysfunction in mouse skeletal muscle. Science.

[37] Brack AS (2007). Increased Wnt signaling during aging alters muscle stem cell fate and increases fibrosis. Science.

[38] Conboy IM (2005). Rejuvenation of aged progenitor cells by exposure to a young systemic environment. Nature.

[39] Hu L (2006). Macrophage low-density lipoprotein receptor-related protein deficiency enhances atherosclerosis in ApoE/LDLR double knockout mice. Arterioscler. Thromb. Vasc. Biol..

[40] Overton CD, Yancey PG, Major AS, Linton MF, Fazio S (2007). Deletion of macrophage LDL receptor-related protein increases atherogenesis in the mouse. Circ. Res..

[41] Rastegarlari G (2012). Macrophage LRP1 contributes to the clearance of von Willebrand factor. Blood.

[42] Muratoglu SC (2011). Macrophage LRP1 suppresses neo-intima formation during vascular remodeling by modulating the TGF-beta signaling pathway. PLoS ONE.

[43] Farr JN (2017). Targeting cellular senescence prevents age-related bone loss in mice. Nat. Med..

[44] van Deursen JM (2014). The role of senescent cells in ageing. Nature.

[45] Jun JI, Lau LF (2010). The matricellular protein CCN1 induces fibroblast senescence and restricts fibrosis in cutaneous wound healing. Nat. Cell Biol..

[46] Curuvija I (2017). Sex differences in macrophage functions in middle-aged rats: relevance of estradiol level and macrophage estrogen receptor expression. Inflammation.

[47] Klionsky DJ, Eskelinen EL, Deretic V (2014). Autophagosomes, phagosomes, autolysosomes, phagolysosomes, autophagolysosomes wait, I’m confused. Autophagy.

[48] Ponpuak M (2015). Secretory autophagy. Curr. Opin. Cell Biol..

[49] Narita M (2011). Spatial coupling of mTOR and autophagy augments secretory phenotypes. Science.

[50] Kozlova N, Jensen JK, Chi TF, Samoylenko A, Kietzmann T (2015). PAI-1 modulates cell migration in a LRP1-dependent manner via beta-catenin and ERK1/2. Thromb. Haemost..

[51] Yamamoto K, Takeshita K, Saito H (2014). Plasminogen activator inhibitor-1 in aging. Semin. Thromb. Hemost..

[52] Amit I, Winter DR, Jung S (2016). The role of the local environment and epigenetics in shaping macrophage identity and their effect on tissue homeostasis. Nat. Immunol..

[53] Lavin Y (2014). Tissue-resident macrophage enhancer landscapes are shaped by the local microenvironment. Cell.

[54] Gruber R (2006). Fracture healing in the elderly patient. Exp. Gerontol..

[55] Ruckh JM (2012). Rejuvenation of regeneration in the aging central nervous system. Cell Stem Cell.

[56] Alexander KA (2011). Osteal macrophages promote in vivo intramembranous bone healing in a mouse tibial injury model. J. Bone Miner. Res..

[57] O’Brien J, Martinson H, Durand-Rougely C, Schedin P (2012). Macrophages are crucial for epithelial cell death and adipocyte repopulation during mammary gland involution. Development.

[58] Schwertfeger KL (2006). A critical role for the inflammatory response in a mouse model of preneoplastic progression. Cancer Res..

[59] Rohlmann A, Gotthardt M, Hammer RE, Herz J (1998). Inducible inactivation of hepatic LRP gene by cre-mediated recombination confirms role of LRP in clearance of chylomicron remnants. J. Clin. Invest..

[60] Amini-Nik S (2014). beta-catenin-regulated myeloid cell adhesion and migration determine wound healing. J. Clin. Invest..

[61] Baht GS, Nadesan P, Silkstone D, Alman BA (2017). Pharmacologically targeting beta-catenin for NF1 associated deficiencies in fracture repair. Bone.

[62] Weischenfeldt J, Porse B (2008). Bone marrow-derived macrophages (BMM): isolation and applications. CSH Protoc..

[63] Yoon C (2013). Low-density lipoprotein receptor-related protein 1 (LRP1)-dependent cell signaling promotes axonal regeneration. J. Biol. Chem..

[64] Nam D (2012). T-lymphocytes enable osteoblast maturation via IL-17F during the early phase of fracture repair. PLoS ONE.

[65] Kramer G, Kasper PT, de Jong L, de Koster CG (2011). Quantitation of newly synthesized proteins by pulse labeling with azidohomoalanine. Methods Mol. Biol..

