# Supplementary material for: Macrophage cells secrete factors including LRP1 that orchestrate the rejuvenation of bone repair in mice
Source: Nat Commun. 2018 Dec 5;9:5191. doi: 10.1038/s41467-018-07666-0 (PMC6281653; doi:10.1038/s41467-018-07666-0)
Supplement: Supplementary file 1 — Supplementary Information [file 41467_2018_7666_MOESM1_ESM.pdf]

Supplementary Information

Macrophage cells secrete factors including LRP1 that orchestrate the rejuvenation of bone repair in mice

Linda Vi†[1,2,] Gurpreet S. Baht†[1,3], Erik J. Soderblom[4], Heather Whetstone[2], Qingxia Wai[2], Bridgette Furman[1], Vijitha Puviindran[1], Puviindran Nadesan[1], Matthew Foster[4], Raymond Poon[2], James P. White[3], Yasuhito Yahara[1], Adeline Ng[5], Tomasa Barrientos[1], Marc Grynpas[5], M. Arthur Mosely[4], Benjamin A. Alman*[1,6]

1 Department of Orthopaedic Surgery, Duke University

2 Hospital for Sick Children, University of Toronto

3 Duke Molecular Physiology Institute, Duke University

4 Proteomics and Metabolomics Shared Resource, Duke University

5 Mount Sinai Hospital, University of Toronto

6 Regeneration Next, Duke University

† These authors contributed equally

Supplementary Table 1. Proteins that were unregulated by at least a twofold change or downregulated by at least 1.5 fold change in young versus old samples.

Protein Description	Fold change Young vs Old
glutaminyl-peptide cyclotransferase	3.909840098
granulin	3.316079451
V-type proton ATPase subunit S1	3.174150297
lysosomal protective protein isoform a	3.099948932
palmitoyl-protein thioesterase 1	3.072714436
meteorin-like protein	3.036030695
beta-glucuronidase	2.903230233
cathepsin Z	2.823707572
gamma-glutamyl hydrolase	2.75361226
cathepsin L1	2.676884047
motif chemokine 9	2.676075195
complement C1q subcomponent subunit A	2.615840423
transcobalamin-2	2.56690318
complement C1q subcomponent subunit B	2.519163311
renin receptor	2.473721227
low-density lipoprotein receptor-related protein 1	2.417881578
beta-2-microglobulin	2.39621159
protein CREG1	2.38996828
macrophage colony-stimulating factor 1 receptor	2.37218178
cathepsin B	2.370927644
H-2 class I histocompatibility antigen, K-W28 alpha chain isoform 1	2.362999472
complement C1q subcomponent subunit C	2.337500678
lysosomal alpha-mannosidase	2.301691452
ref| 45 kDa calcium-binding protein	2.264816857
stabilin-1	2.246209351
sulfated glycoprotein 1 isoform A	2.182756329
platelet-activating factor acetylhydrolase	2.153066186
beta-hexosaminidase subunit alpha	2.08830909
cystatin-C	2.063974018
galectin-3-binding protein	2.010385692
transmembrane gamma-carboxyglutamic acid protein 2	-1.579377062
fibrillin-1	-1.587303575
collagen alpha-1(I) chain	-1.68889359
periostin isoform 2	-1.754342941
plasminogen activator inhibitor 1	-2.286915157
collagen alpha-2(I) chain	-2.745622638

Supplementary Figures

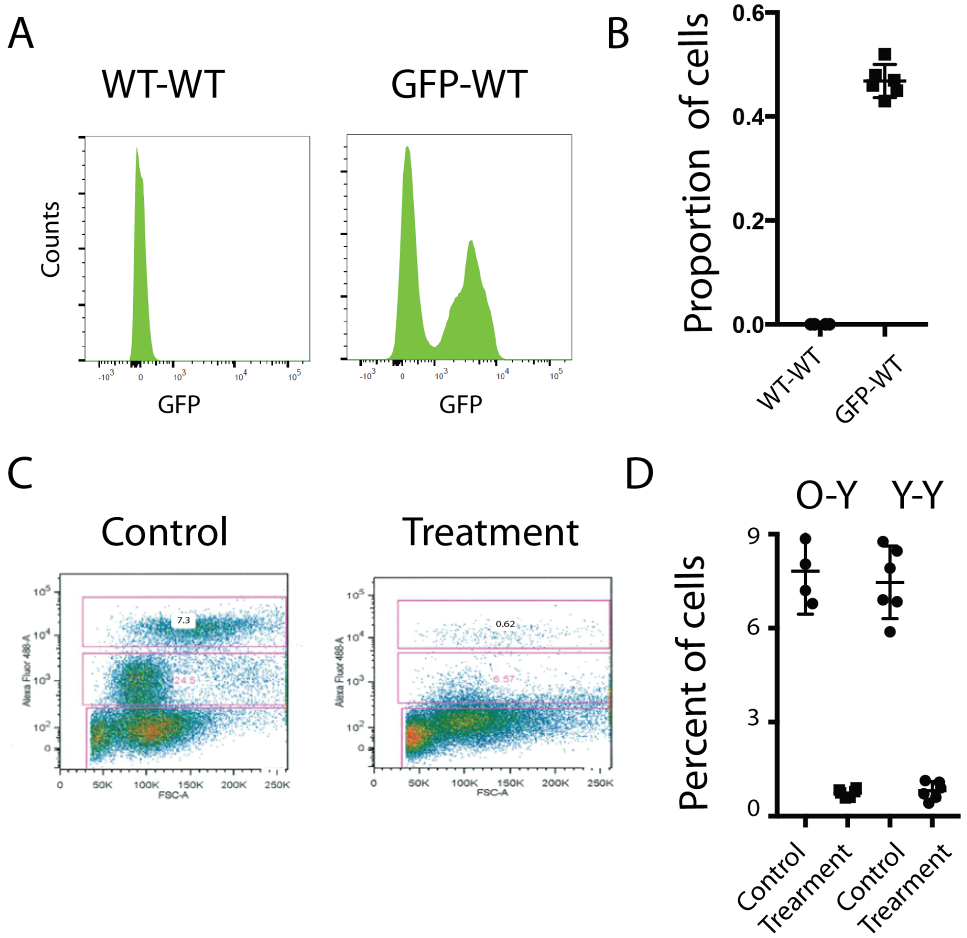

Supplementary Figure One

Supplementary Fig. 1: Parabiotic pairs share blood supply and AP20187effective depletes macrophage cells in MaFIA mice. A) A representative flow cytometry analysis from parabiotic pairs, using either two wild type mice (WT-WT), or one mouse expressing GFP and the other a wild-type mouse (GFP-WT). B) Data showing proportion of GFP expressing cells as deteetd using flow cytometry analysis from parabiotic pairs of either two wild type mice (WT-WT), or one mouse expressing GFP and the other a wild-type mouse (GFP-WT).own as mean and 95% confidence intervals. The GFP-WT pairs show sharing of blood supply with between 45 and 52% of macrophage cells in the GFP-WT pair expressing GFP. C) Representative Flow analysis showing that AP20187 treatment reduces the proportion of GFP expressing cells in the hematopoietic cell population from MaFIA mice (macrophage cells) in parabiotic pairs. The top box is the GFP expressing cells. D) The percentage of cells expressing GFP in the various pairs examined is given as a data point for the each parabiotic pair tested, and the mean and 95% confidence interval are shown for each experimental group. There was no difference in the ability of AP20187 to deplete macrophage cells (express GFP) between parabiotic pairs of old-young (O-Y) or young-young (Y-Y) mice in which one of the young mice was a MaFIA mouse. Greater than 90% of macrophages are depleted in each instance. Comparison using paired t-test.

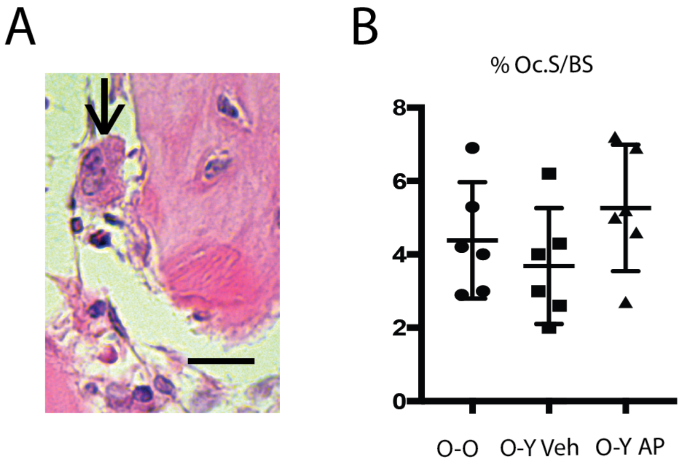

Supplementary Figure Two

Supplementary Fig. 2: Osteoclast numbers in experimental parabiosis groups. A) A representative histologic section from a parabiotic pair treated with AP20187 is shown. The arrow indicates an osteoclast. The size bar is 250um long. B) Parabiotic pairs of old-old (O-O) or old-young (O-Y) mice, in which the young mice are MaFIA mice. AP indicates pairs treated with the dimerizing agent, and Veh indicates mice treated with carrier. The percentage of osteoclast surface to bone surface (Oc.S/BS) for each experimental parabiotic group is shown (n=6 for each group) as means and 95% confidence intervals. There is no significant difference observed between groups. Comparison using ANOVA.

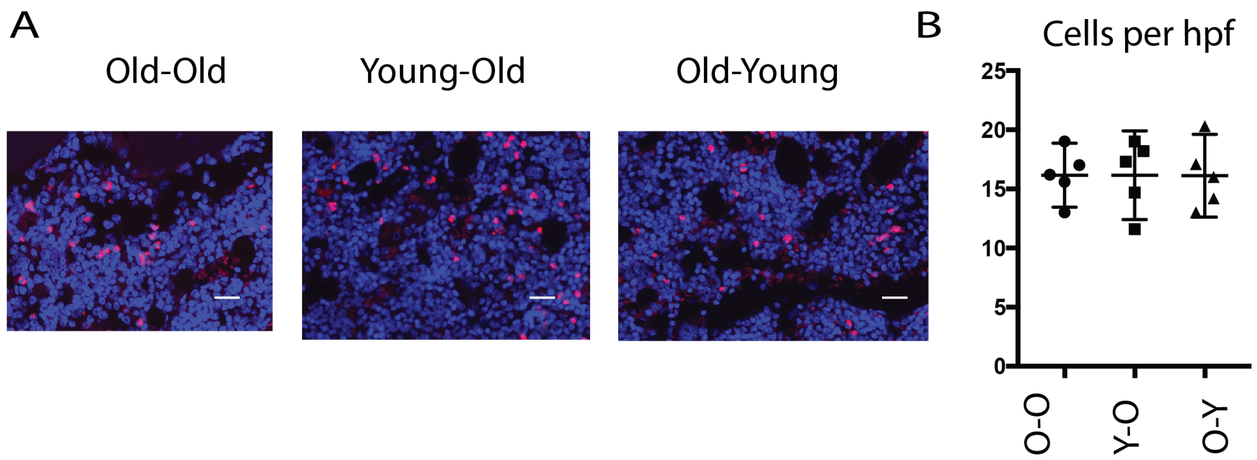

Supplementary Figure Three

Supplementary Fig. 3: Macrophage numbers in various experimental groups in which bone marrow transplantation was performed. A) Representative histologic sections from the bone marrow at the fracture site. Fluorescent cells are the GFP expressing macrophage cells from MaFIA mice donors that were not treated with the dimerizing agent. The age pairs indicate the age of the donor and recipient mouse, with the donor mouse listed first. O = old, and Y= young. The blue color is DAPI stained nuclei. The size marker is 200um. B) The data point showing the number of fluorescent cells per high powered field for each animal examined, and the mean and 95% confidence interval for each experimental group (data and n given in the figure with n= 5). There are no differences in numbers of cells between the groups. Comparison using ANOVA.

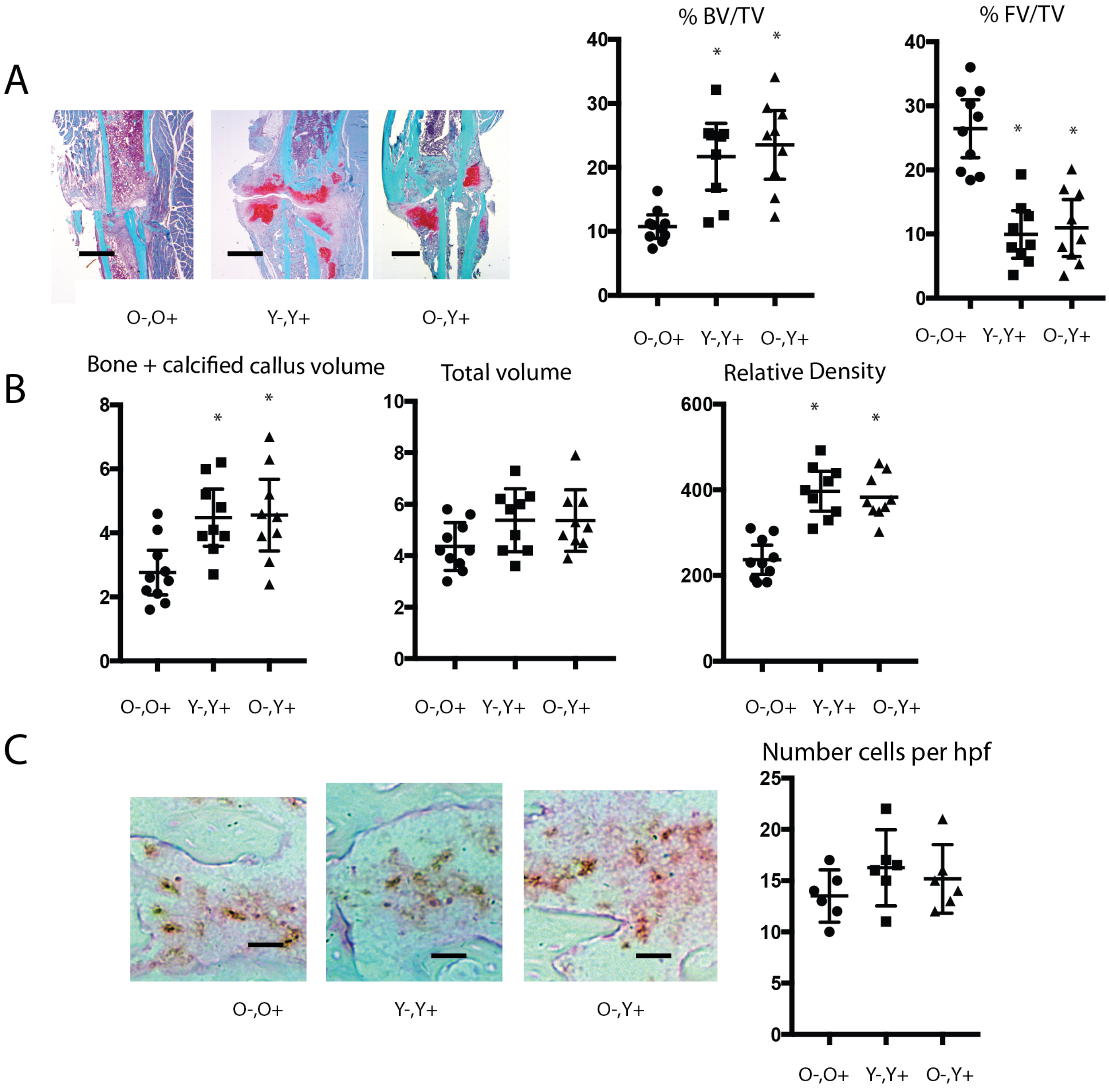

Supplementary Figure Four

Supplementary Fig. 4: Fracture healing characteristics after two weeks in old mice transplanted with macrophage cells from either old or young mice. A) Safranin O staining histologic sections two weeks following fracture and associated histomorphometric analysis. n=10 for O-,O+; 9 for Y,-Y+; and 9 for O-, Y+. Graphs show bone volume/total volume (BV/TV) in %; total fibrous tissue/total volume (FV/TV) in % means and 95% confidence intervals. An asterisk indicates a significant difference ($p<0.05$) compared to the O-,O+ group. B) Analysis of micro-CT data from the same animals. Data given as bone plus calcified callus volume (mm^3), total callous volume (mm^3), or relative density. Data given as mean and 95% confidence intervals. An asterisk indicates a significant difference ($p<0.05$) compared to the O-,O+ group. C) Representative histologic sections showing F4/80 stained cells in the three groups at the site of the fracture. Numbers of cells per high powered field are shown for each experimental group (n=6 for each group). O = old, Y= young. BV = bone volume, TV = total volume, and FV = fibrous volume. Means and 95% confidence intervals are shown, and there is no significant difference observed between the groups. Comparison using ANOVA.

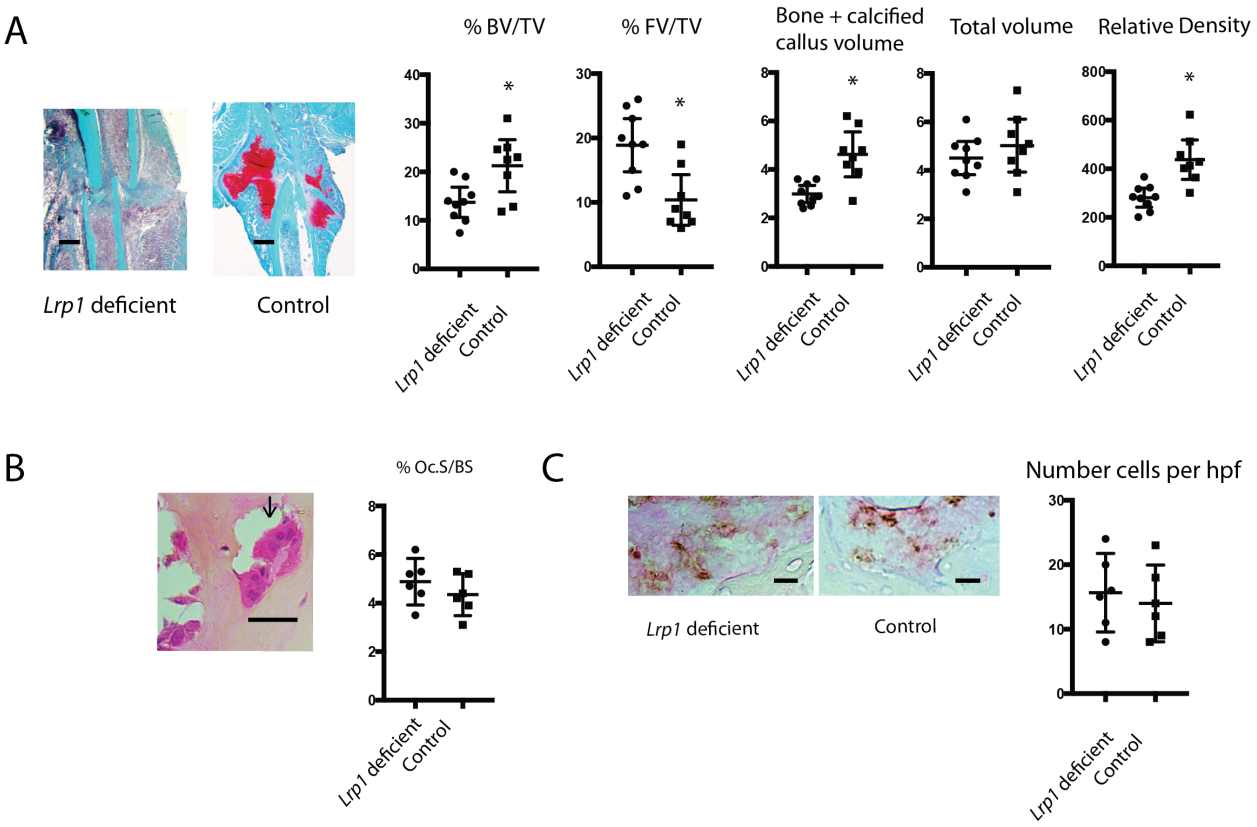

Supplementary Figure Five

Supplementary Fig. 5: Fracture healing at two weeks and osteoclast and macrophage numbers in fractures at four weeks in old mice transplanted with bone marrow cells from young mice lacking *Lrp1* in monocyte/macrophage lineage cells or controls. A) Representative Safranin O staining histologic sections two weeks following fracture and associated histomorphometric and micro-CT data analysis. Size marker in 0.5mm. n=9 for control and 8 for Lrp1 deficient monocyte lineage bone marrow transplant. Data given as bone volume/total volume (BV/TV) in %, total fibrous tissue/total volume (FV/TV) in %, bone plus calcified callus volume (mm^3), total callous volume (mm^3), or relative density. Means and 95% confidence intervals are shown, and an asterisk indicates a significant difference ($p<0.05$) compared to the control group. B) Multinucleated osteoclasts in the mice from each experimental group at the fracture site. A representative histologic section from a mouse transplanted with *Lrp1* deficient monocyte lineage bone marrow cells. An arrow points to an osteoclast. Size marker is 250um. The percentage of osteoclast surface to bone surface (Oc.S/BS) for each experimental group is shown (n=6 for each group), as the means and 95% confidence intervals. There is no significant difference observed between groups. C) Histologic sections showing F4/80 stained cells in the three groups at the site of the fracture. Size marker is 200um. Numbers of cells per high powered field (hpf) are shown for each experimental group (n=6 for each group). Means and 95% confidence intervals are shown, and there is no significant difference between the mice translated with control bone marrow or with *Lrp1* deficient bone marrow. Comparison using paired t-test.

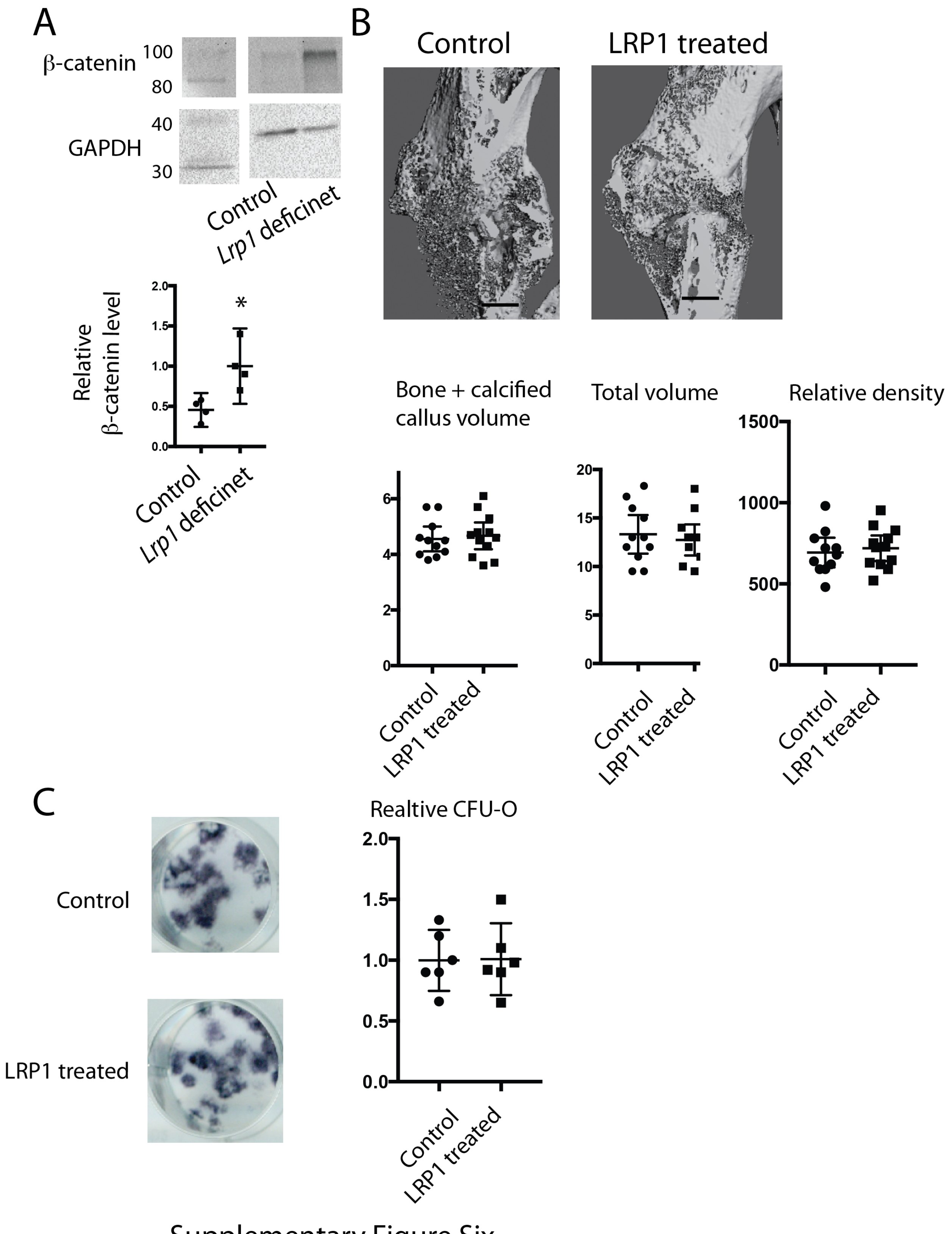

Supplementary Figure Six

Supplementary Fig. 6: β-catenin mediates the effect of Lrp1 on fracture rejuvenation. A) Representative Western blot (top) and individual data points for relative expression for each fracture analyzed, showing that there is a higher β-catenin protein level in fractures from old mice in which bone marrow cells were transplanted from young donor macrophage cells lacking Lrp1. The first lane is the size marker (full blots in supplementary Fig.7). Each data point is shown, along with the mean and 95% confidence interval for each experimental group. An asterisk indicates a significant difference compared to the control group, $p<0.05$. B) Fracture healing at four weeks in old mice homozygous *Catnb*^{tm2Kem} mice, treated with and adenovirus expressing cre-recombinase to deplete β-catenin at the fracture site. Mice were treated with either LRP1 or carrier at the fracture site. Representative micro-CT images at four weeks following the fracture (size bar is 0.5mm), and micro-CT data given as bone plus calcified callus volume (mm^3), total callous volume (mm^3), or relative density. Each data point for each mouse analyzed is shown along with the mean and 95% confidence interval for each experimental group. There is no difference with LRP1 treatment. In mice lacking β-catenin. C) CFU-O from bone marrow from homozygous *Catnb*^{tm2Kem} mice. Treatment with LRP1 did not alter the CFU-O numbers. Each data point is shown along with the mean and 95% confidence interval for each experimental group. Comparison using paired t-test.

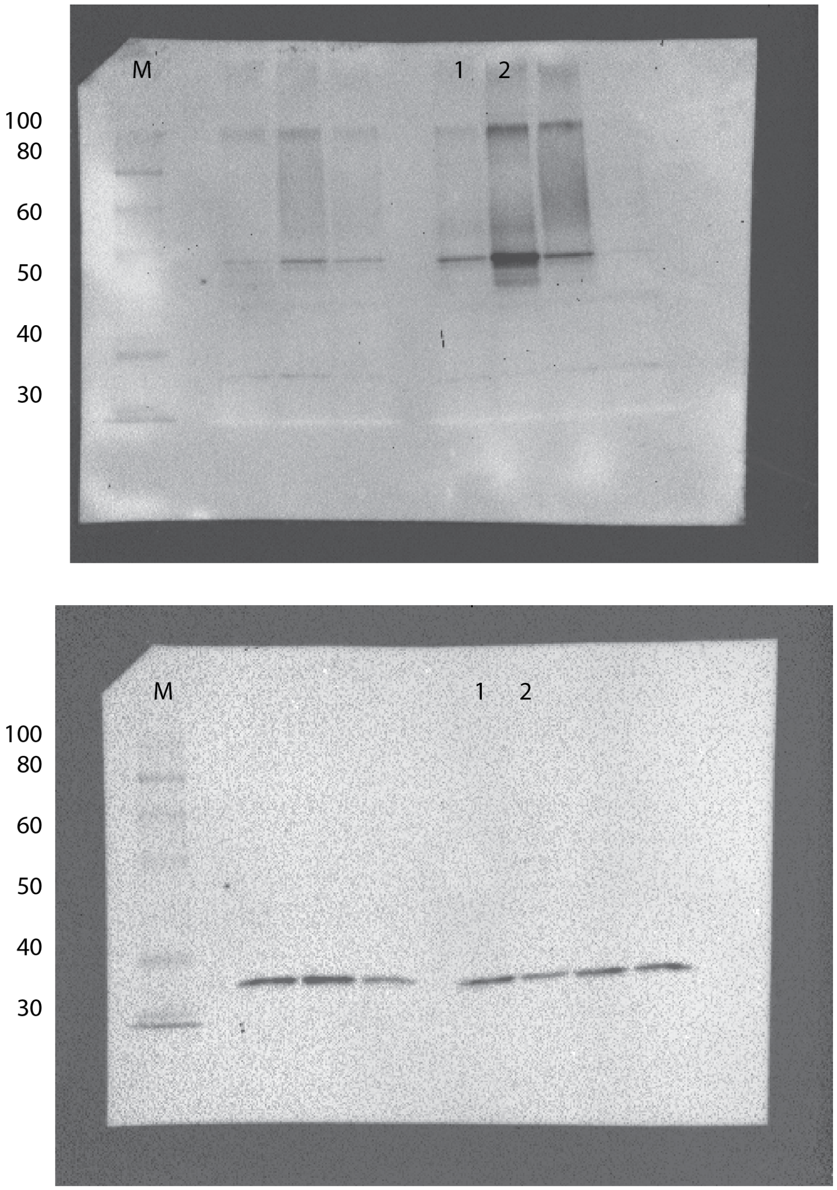

Supplementary Figure Seven

Supplementary Fig. 7: Full western blot shown in supplementary Fig. 6 panel A. Top panel shows blot using an antibody to beta-catenin and bottom panel shows a blot using an antibody to GAPDH as a loading control. Lane labeled "M" is the size marker. Lanes labeled '1" and "2" are the data shown in Supplementary Fig. 6.
